# Altitudinal Visual Field Defect as the Presenting Sign of a Brain Tumor: A Case Report

**DOI:** 10.1002/ccr3.73451

**Published:** 2026-09-09

**Authors:** Farid Shekarchian, Mehrdad Motamed Shariati, Mohammadreza Same’

**Affiliations:** ^1^ Eye Research Center Mashhad University of Medical Sciences Mashhad Iran

**Keywords:** altitudinal visual, brain tumor, tumor, visual field

## Abstract

Brain tumors present with symptoms depending on their type, location, and stage. Most cause visual field defects respecting vertical meridians. Compressive optic neuropathy may produce altitudinal defects, mimicking ischemic optic neuropathy. Therefore, brain tumors should be included in the differential diagnosis, as early detection may improve clinical outcomes.

## 1 Introduction

A brain tumor occurs when a group of cells within the brain become cancerous and grow uncontrollably, forming a mass. Brain tumors are among the most malignant types of tumors in humans, accounting for approximately 1.35% of all malignant neoplasms and 29.5% of cancer‐related deaths [1]. The uncontrolled proliferation of abnormal brain tissue increases intracranial pressure, leading to compression of adjacent neural structures and disruption of normal cerebral function. Depending on the tumor's size, location, and growth rate, patients may develop a wide range of neurological manifestations, including headaches, seizures, cognitive impairment, focal neurological deficits, and visual disturbances due to involvement of the optic pathways or elevated intracranial pressure [2].

Brain tumors are among the most challenging neurological disorders due to their complex biological characteristics, high mortality rates, and significant impact on patients' quality of life. They are broadly classified into two major categories: benign (noncancerous) and malignant (cancerous) tumors [3].

Benign tumors are generally characterized by slow growth, well‐defined borders, and limited invasion into surrounding brain tissues. Despite their noncancerous nature, benign tumors may still cause severe neurological deficits by compressing adjacent brain structures or increasing intracranial pressure, depending on their size and anatomical location [4].

In contrast, malignant brain tumors exhibit aggressive biological behavior, including rapid cellular proliferation, diffuse infiltration into healthy brain tissues, and a high tendency for recurrence following treatment. These characteristics make malignant tumors substantially more difficult to treat and are associated with significantly poorer clinical outcomes. Furthermore, certain malignant tumors may disseminate through the cerebrospinal fluid or metastasize to other regions of the central nervous system, further complicating disease management [5].

The early and accurate diagnosis of brain tumors is a critical factor in determining appropriate therapeutic strategies and improving patient prognosis. Timely detection enables clinicians to initiate suitable interventions, including surgical resection, radiotherapy, chemotherapy, or targeted therapies, before the disease progresses to advanced stages [6]. Numerous clinical studies have demonstrated that early diagnosis contributes to increased survival rates, reduced treatment‐related complications, and improved neurological outcomes. However, distinguishing between different tumor types and grades remains a challenging task because of the considerable variability in tumor morphology, size, location, texture, and imaging characteristics across patients. These challenges emphasize the need for reliable and objective diagnostic methods capable of supporting clinical decision‐making [7].

The World Health Organization (WHO) classifies primary brain tumors into four histopathological grades (I–IV) according to their cellular morphology, mitotic activity, vascular proliferation, and necrosis. Grade I tumors are typically slow‐growing, well‐circumscribed lesions with favorable prognoses and are often curable through complete surgical excision. Grade II tumors exhibit relatively slow progression but possess infiltrative characteristics and may transform into higher‐grade malignancies over time. Grades III and IV are categorized as high‐grade gliomas, representing the most aggressive forms of primary brain tumors. These tumors are characterized by rapid growth, increased mitotic activity, diffuse infiltration into surrounding brain tissues, extensive angiogenesis, and a high probability of recurrence despite multimodal treatment approaches. Among them, Grade IV glioblastoma is recognized as the most aggressive and lethal primary brain tumor, with a median survival time of approximately 12–18 months even after maximal surgical resection followed by concurrent chemoradiotherapy [8, 9].

Given the substantial differences in biological behavior, therapeutic management, and clinical outcomes among various tumor grades, accurate tumor classification plays a pivotal role in modern neuro–oncology. Precise grading facilitates personalized treatment planning, prognosis estimation, postoperative monitoring, and assessment of therapeutic response. Consequently, there has been increasing interest in developing computer‐aided diagnosis (CAD) systems based on advanced medical imaging and artificial intelligence techniques to enhance diagnostic accuracy, reduce interobserver variability, and assist radiologists in the interpretation of brain magnetic resonance imaging (MRI) [10].

These automated approaches have demonstrated considerable potential in improving the efficiency and reliability of brain tumor detection, segmentation, grading, and classification, thereby contributing to more effective clinical decision‐making and improved patient care. Diagnosing and treating brain tumors is particularly challenging due to the wide variations in tumor shape, appearance, size, location, associated symptoms, and imaging parameters. Common symptoms include headache, nausea, vomiting, altered mental status, papilledema, seizures, and visual disturbances. Visual symptoms can range from mild blurred vision in the early stages to severe vision loss or blindness in the advanced stages [11]. Prompt diagnosis and treatment are essential to prevent permanent brain damage or fatal outcomes.

Brain tumors are typically diagnosed using CT scans and MRIs. Ophthalmic evaluations, including visual acuity tests, relative afferent pupillary defect (RAPD) assessments (Marcus Gunn test), eye movement tests, anterior and posterior segment examinations, and optic nerve evaluations, can help detect visual symptoms. Additional diagnostic tools such as perimetry and optic nerve head imaging can further aid in evaluating visual field defects [12].

Treatment approaches vary based on the tumor type and stage. While surgery is often the primary treatment in advanced cases, other options such as chemotherapy, radiation therapy, and virotherapy are available for earlier‐stage tumors [13].

In this case report, we introduce a middle‐aged woman with progressive vision loss who showed altitudinal visual field defects in her perimetry test, which finally led to the diagnosis of a benign brain tumor. The patient subsequently underwent surgical resection, during which the brain tumor was removed. Histopathological examination was performed to confirm the diagnosis and determine the tumor grade. Following surgery, the patient received adjuvant chemotherapy according to the standard treatment protocol and was closely monitored through periodic clinical evaluations and follow‐up MRI examinations to assess therapeutic response and detect any evidence of tumor recurrence.

## 2 Case History

A 39‐year‐old woman was referred to the ophthalmic emergency department with complaints of blurred vision, headache, and tinnitus that had persisted for 2 months.

## 3 Methods (Investigations and Treatment)

Ophthalmologic examination revealed a visual acuity of 10\10 in the right eye (OD) and 9\10 in the left eye (OS). A + 1 relative afferent pupillary defect (RAPD) (Marcus Gunn sign) was observed in the left eye. RAPD occurs when one eye responds less vigorously to light than the other, indicating asymmetric function of the optic nerves or retina. Grading of RAPD (commonly from +1 to +4): +1 RAPD: Mild defect: There is an initial weak constriction followed by dilation when the light is moved to the affected eye. +2 to +3 RAPD: Moderate defect: Immediate dilation with little or no initial constriction. +4 RAPD: Severe defect: Immediate and full dilation, often associated with no light perception in that eye. RAPD +1 (Relative Afferent Pupillary Defect, Grade +1) refers to a mild defect in the afferent (sensory) pathway of the pupillary light reflex, usually due to optic nerve or severe retinal disease in one eye. Also it is defined as 1 RAPD (Marcus Gunn phenomenon) is defined as a pupillary response where the affected pupil constricts initially but undergoes early redilation (escape), rather than holding the constriction, when the light source is swung from the normal to the affected eye.

Anterior and posterior segment examinations were unremarkable. The intraocular pressure (IOP) and eye movements were within normal limits for both eyes. We ordered optic nerve head (ONH) and ganglion cell complex (GCC) optical coherence tomography (OCT) for further evaluation.

**FIGURE 1:**
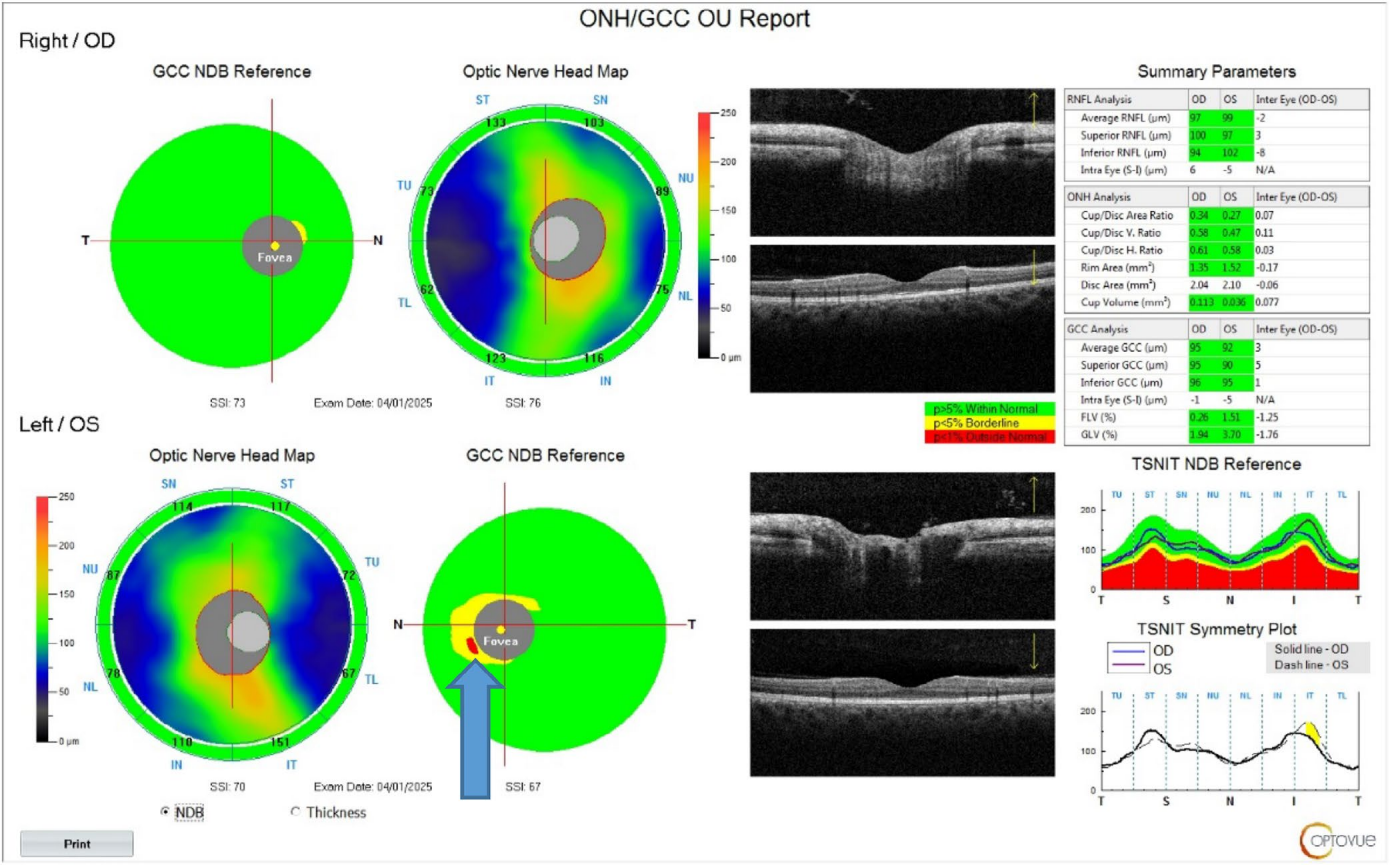
Optical coherence tomography (OCT) scan of the ONH/GCC, revealing mild attenuation of the ganglion cell complex in the left eye. The arrow denotes the area of interest/lesion. Imaging performed using an Optovue OCT system.

Optical nerve head (ONH) and GCC imaging showed mild ganglion cell complex loss in the left eye (Figure 1).

**FIGURE 2:**
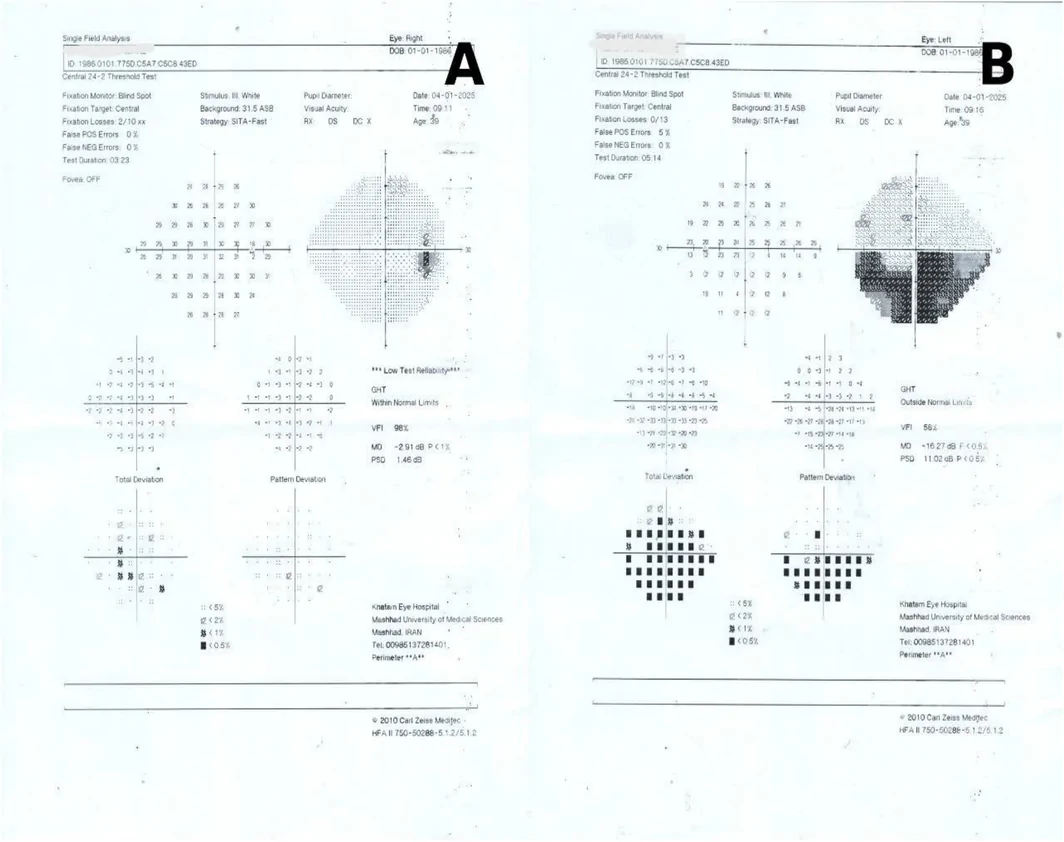
Humphrey automated perimetry of both eyes in patient. (A) Right eye perimetry is in standard limit. (B) Left eye perimetry is abnormal with altitudinal visual field defect and horizontal respect.

Visual field testing showed an altitudinal inferior visual field defect, respecting the horizontal line (Figure 2), compatible with the diagnosis of optic nerve vascular disorders like posterior ischemic optic neuropathy. However, the differential diagnosis of vascular diseases did not justify other symptoms like persistent headaches and tinnitus.

**FIGURE 3:**
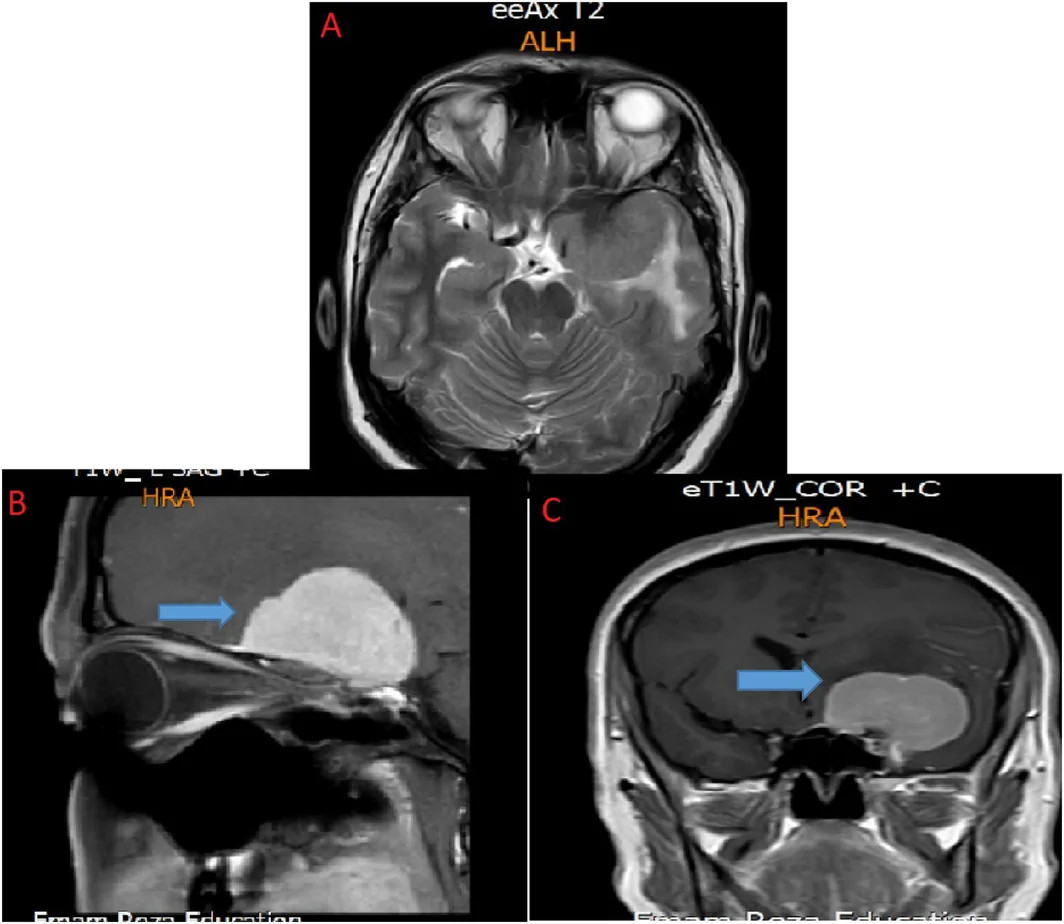
(A) Axial T2 MRI without contrast and left retrobulbar mass. (B) Sagittal T1 MRI with contrast and fat suppression and orbital apex mass enhancement. (C) Coronal T1 MRI with contrast clinoidal enhanced mass (the arrows point to the tumor).

As the next step, a brain and orbit MRI was ordered, revealing an unexpected brain tumor (Figure 3).

## 4 Conclusions and Results

Following consultation with a neurosurgery specialist, the patient was immediately referred to the neurosurgery department for further treatment. Ultimately, the patient was diagnosed with a meningioma.

Following the clinical presentation of progressive visual blurring, persistent headache, and tinnitus, the patient was promptly referred for neurosurgical evaluation. The patient underwent surgical intervention for tumor resection, which was subsequently followed by postoperative adjuvant radiotherapy. Histopathological analysis confirmed the diagnosis of a benign (WHO Grade I) meningioma. Notably, this comprehensive therapeutic approach resulted in the successful preservation of the patient's baseline visual acuity. To monitor for any potential recurrence or disease progression, the patient remains under active surveillance with regular periodic brain MRI follow‐ups, demonstrating stable neurological status to date.

## 5 Discussion

Brain tumors can present with a variety of visual and neurological symptoms, depending on their type, location, and stage. Visual field defects are among the most common ophthalmic manifestations, often respecting the vertical meridian due to the anatomical organization of the visual pathways. However, in some cases, brain tumors may cause compressive optic neuropathy, leading to altitudinal visual field defects—typically associated with ischemic optic neuropathy—making an accurate differential diagnosis essential [14].

The differential diagnosis of brain tumors includes a range of neoplastic and nonneoplastic conditions that can affect the visual pathways and optic nerve. Meningiomas, which arise from the meninges, are among the most common intracranial tumors affecting the optic nerve and chiasm. They often cause gradual, progressive visual loss and optic disc pallor, with characteristic imaging findings such as a dural tail on MRI. Optic nerve sheath meningiomas can compress the optic nerve, leading to chronic visual deterioration with optic atrophy [15].

Gliomas, another important differential diagnosis, can be classified into low‐grade and high‐grade types. Optic pathway gliomas, often seen in children and associated with neurofibromatosis type 1, typically present with slowly progressive visual loss and optic disc changes. In contrast, glioblastomas (glioblastoma multiforme) are highly aggressive tumors that can cause rapid neurological deterioration, often with significant mass effect and edema leading to visual disturbances, headaches, and other neurological deficits [16].

Craniopharyngiomas, benign but locally invasive tumors arising from remnants of Rathke's pouch, frequently involve the optic chiasm and hypothalamus. These tumors are a well‐known cause of bitemporal hemianopia due to compression of the chiasm. In addition to visual field defects, patients may present with endocrine dysfunction due to involvement of the pituitary gland [17].

Other brain tumors that can affect the visual pathways include pituitary adenomas, which can cause bitemporal hemianopia or junctional scotomas when compressing the optic chiasm. Hemangioblastomas, often associated with von Hippel–Lindau disease, can affect the cerebellum and brainstem, sometimes leading to visual symptoms due to increased intracranial pressure. Metastatic brain tumors should also be considered in patients with known primary malignancies, as they can produce compressive effects on the visual pathways [7, 9].

Distinguishing between these tumors and other causes of optic neuropathy, such as ischemic optic neuropathy, requires a comprehensive clinical and imaging evaluation. While ischemic optic neuropathy typically presents with acute, painless vision loss and disc edema, compressive optic neuropathy due to tumors tends to follow a more insidious course, often with progressive worsening of visual function and optic disc pallor. MRI with contrast remains the gold standard for identifying intracranial tumors affecting the optic nerve, chiasm, or retrochiasmal pathways [11].

Early recognition of compressive optic neuropathy due to brain tumors is crucial to prevent irreversible visual loss. In clinical practice, patients presenting with unexplained or progressive visual field defects—particularly those with atypical patterns such as altitudinal or arcuate deficits not fully accounted for by vascular or glaucomatous causes—should prompt consideration of intracranial space‐occupying lesions, including meningiomas, pituitary adenomas, and other parasellar or sphenoid wing tumors.

Compressive lesions may initially mimic more common causes of optic neuropathy, leading to delays in diagnosis. Unlike ischemic optic neuropathy, which often has a sudden onset, compressive neuropathies typically present with a more insidious progression, preserved optic disc appearance in early stages, and are sometimes associated with systemic or neurological symptoms such as headaches, tinnitus, or hormonal disturbances.

Timely neuroimaging, especially MRI with contrast, plays a pivotal role in detecting structural lesions affecting the anterior visual pathway. Early identification enables prompt neurosurgical or medical intervention, which may halt or even reverse visual deterioration, depending on the duration and extent of optic nerve compression.

Therefore, a high index of suspicion and a multidisciplinary approach involving ophthalmologists, neurologists, and neurosurgeons is essential, particularly in patients with progressive or atypical visual symptoms. This can significantly improve both visual prognosis and overall neurological outcomes.

## Author Contributions

**Farid Shekarchian:** conceptualization, supervision, writing – review and editing. **Mehrdad Motamed Shariati:** supervision, writing – original draft, writing – review and editing. **Mohammadreza Same’:** supervision, visualization, writing – review and editing.

## Funding

The authors have nothing to report.

## Consent

Written informed consent was obtained from the patient to publish this report in accordance with the journal's patient consent policy.

## Conflicts of Interest

The authors declare no conflicts of interest.

## Data Availability

The data that support the findings of this study are available on request from the corresponding author. The data are not publicly available due to privacy or ethical restrictions.
